# TARG1 affects EGFR signaling through the regulation of RNA metabolism

**DOI:** 10.1038/s41598-025-08010-5

**Published:** 2025-07-02

**Authors:** Mihály Mérey, Roberta Fajka-Boja, Gergely Imre, Péter Gudmann, Zsolt Török, Lajos Mátés, Ágnes Czibula, Gyula Timinszky

**Affiliations:** 1 Laboratory of DNA Damage and Nuclear Dynamics, Institute of Genetics, HUN-REN Biological Research Centre, Szeged, 6726 Hungary; 2 Doctoral School of Multidisciplinary Medical Sciences, University of Szeged, Szeged, 6720 Hungary; 3 Department of Immunology, University of Szeged, Szeged, 6720 Hungary; 4 Laboratory of Cancer Genome Research, Institute of Genetics, HUN-REN Biological Research Centre, Szeged, 6726 Hungary; 5 Doctoral School of Biology, University of Szeged, Szeged, 6720 Hungary; 6 Laboratory of Molecular Stress Biology, Institute of Biochemistry, HUN-REN Biological Research Centre, Szeged, 6726 Hungary

**Keywords:** RNA transport, PolyADP-ribosylation, Cell migration, Cell signalling, Cell biology, PolyADP-ribosylation

## Abstract

Epidermal Growth Factor Receptor (EGFR) signaling plays a central role in cell proliferation, migration, and survival. Emerging evidence suggests a connection between ADP-ribosylation and EGFR regulation. Previous studies implicated PARP’s role in EGFR signaling, but the participation of ADP(ribosyl)hydrolases in it, that can revert their enzymatic modifications, still remained elusive. The role of TARG1, a macrodomain-containing hydrolase, that has been implicated in RNA metabolism, and cellular stress response, but was not studied in EGFR signaling before. Here, we investigate the impact of TARG1 depletion in U2-OS osteosarcoma cells using knockout (KO) and knockdown (KD) models. We find that TARG1 loss reduces both EGFR protein and mRNA levels. Our results show increased mRNA turnover and altered RNA distribution and translation in TARG1 KO cells, suggesting that TARG1 influences RNA metabolism and translational regulation. Notably, TARG1-deficient cells exhibit heightened sensitivity to MEK1/2 inhibition, indicating potential crosstalk between TARG1 and the Ras/MEK/ERK pathway. These findings suggest that TARG1, and possibly ADP-ribosylation, regulate EGFR expression and translation through RNA biogenesis-mediated mechanisms, highlighting its potential role in cancer cell signaling and survival.

**Supplementary Information:**

The online version contains supplementary material available at 10.1038/s41598-025-08010-5.

## Introduction

Epidermal Growth Factor Receptors (EGFRs) belong to a large family of receptor tyrosine kinases that are overexpressed in many cancer types, including breast, lung, esophageal, head and neck cancers and various types of osteosarcoma as well^1,2^. EGFR and its family members are primarily located on the cell membrane, where they interact with extracellular ligands. This binding triggers conformational changes that activate the receptors, initiating complex signaling cascades that regulate key processes such as growth, differentiation, adhesion, migration, and survival of cancer cells. Due to their crucial role in cancer progression, EGFR and its related receptors have become attractive targets for anti-cancer therapies^3–6^.

Clinically, two main classes of canonical inhibitors are used to counteract the aberrant signaling pathways driven by EGFR overexpression or mutation. The first class includes monoclonal antibodies that target the extracellular ligand-binding domain, competing with natural ligands to prevent receptor activation. The second class consists of small molecule inhibitors, known as tyrosine kinase inhibitors (TKIs), which *cross the cell membrane*, and bind to the ATP-binding pocket within the receptor’s intracellular kinase domain^7–9^. Despite their clinical efficacy, resistance often emerges frequently due to mutations in EGFR that alter its protein structure, rendering the receptor resistant to both monoclonal antibodies and TKIs by concealing the inhibitor binding sites^10,11^.

The emergence of EGFR inhibitor resistance has prompted investigations into combination therapies that target complementary pathways. In 2012, Nowsheen et al. reported that the combined treatment of triple-negative breast cancer cell lines with EGFR and PARP inhibitors resulted in a synthetic lethal interaction both in vitro and in vivo^12^. Another report revealed that PARP1 inhibition suppressed EGFR expression levels and affected downstream pathways, such as Akt, p38, and ERK, which are crucial for cell proliferation and migration^13,14^.

ADP-ribosylation is a widespread reversible modification that occurs across all kingdoms of life, affecting biomolecules such as nucleic acids and various protein amino acid residues. In this process, enzymes transfer ADP-ribosyl moiety from NAD^+^ onto the target molecule, releasing nicotinamide^15^. The ADP-ribose (ADPr) units are covalently attached to specific targets. These modifications vary in length, from single ADPr mono(ADP-ribosylation) to complex poly(ADP-ribose) chains, which can also adopt branched structures. The enzymes responsible for adding ADPr to their targets are known as (ADP-ribosyl)transferases (ARTs), while those that remove the modification are termed (ADP-ribosyl)hydrolase enzymes^16^.

ADP-ribosylation plays a critical role in various cellular processes, most notably in the DNA damage response, but also in chromatin remodeling, transcription regulation, and RNA processing^17^. In mammals, 17 members of the (ADP-ribosyl)transferase superfamily have been identified, commonly referred to as PARPs^18^. Some PARPs, such as PARP1, PARP2, Tankyrase-1, and Tankyrase-2, generate poly(ADP-ribose) chains (up to 200 units) linked by unique *O*-glycosidic ribose-ribose bonds, a process known as poly(ADP-ribosyl)ation (PARylation)^19^. However, the majority of human PARP members are involved in mono(ADP-ribosyl)ation (MARylation), which involves the transfer of a single ADPr unit to target proteins^20^. Both PARylation and MARylation are key regulators of cellular functions and responses, highlighting the importance of ADP-ribosylation in maintaining cellular homeostasis.

Two distinct protein families exhibit hydrolytic activity against ADP-ribosylated proteins, targeting a broad range of substrates: the (ADP-ribosyl)hydrolases (ARH) and the macrodomain-containing enzymes. The ARH family of proteins (ARH1-3) have similar size (39 kDa) and amino acid sequence. ARH1 is a mono(ADP-ribosyl)-arginine hydrolase, which catalyzes the hydrolysis of the *N*-glycosidic bond linking ADP-ribose to the guanidino group of arginine, leading to release of ADP-ribose, with formation of arginine. ARH3 catalyzes the removal of ADP-ribose moieties from serine^21^.

Macrodomain-containing proteins are characterized by a conserved ADP-ribose recognition domain known as the macrodomain. The macrodomain is crucial for the binding and hydrolysis of ADPr modifications across various cellular compartments^22^. The presence of macrodomains in vertebrates, as well as in a wide array of bacteria, archaea, viruses, and plants, underscores their evolutionary conservation and functional significance^23,24^. In mammalian cells three macrodomain-containing proteins—MACROD1, MACROD2, and TARG1—have mono(ADP-ribosyl)hydrolase activity^25^. These enzymes are involved in deacetylating *O-*acetyl-ADPr^26^, removing mono-ADPr from modified proteins^27^, and cleaving ADPr from mono(ADP-ribosyl)ated (MARylated) DNA and RNA in vitro^28–30^. Of these, TARG1 localizes predominantly to nucleoli, with additional presence in the nucleoplasm and weaker signals in the cytoplasm. In U2-OS cells lacking TARG1, an increase in both the number of nucleoli and the total nucleolar area was observed, suggesting a potential role for TARG1 in nucleolar dynamics^31^. A BioID experiment conducted by Žaja et al. identified several interacting proteins with TARG1, suggesting that in addition to its localization in stress granules, TARG1 may also be involved in nucleolar and possibly cytoplasmic RNA metabolism^31^.

Given the potential interplay between ADP-ribosylation and EGFR signaling^32^, we aimed to explore the role of TARG1 in modulating EGFR expression and its impact on cancer cell behavior.

## Results

### Cell migration is impaired in the TARG1 knockouts

**Fig. 1:**
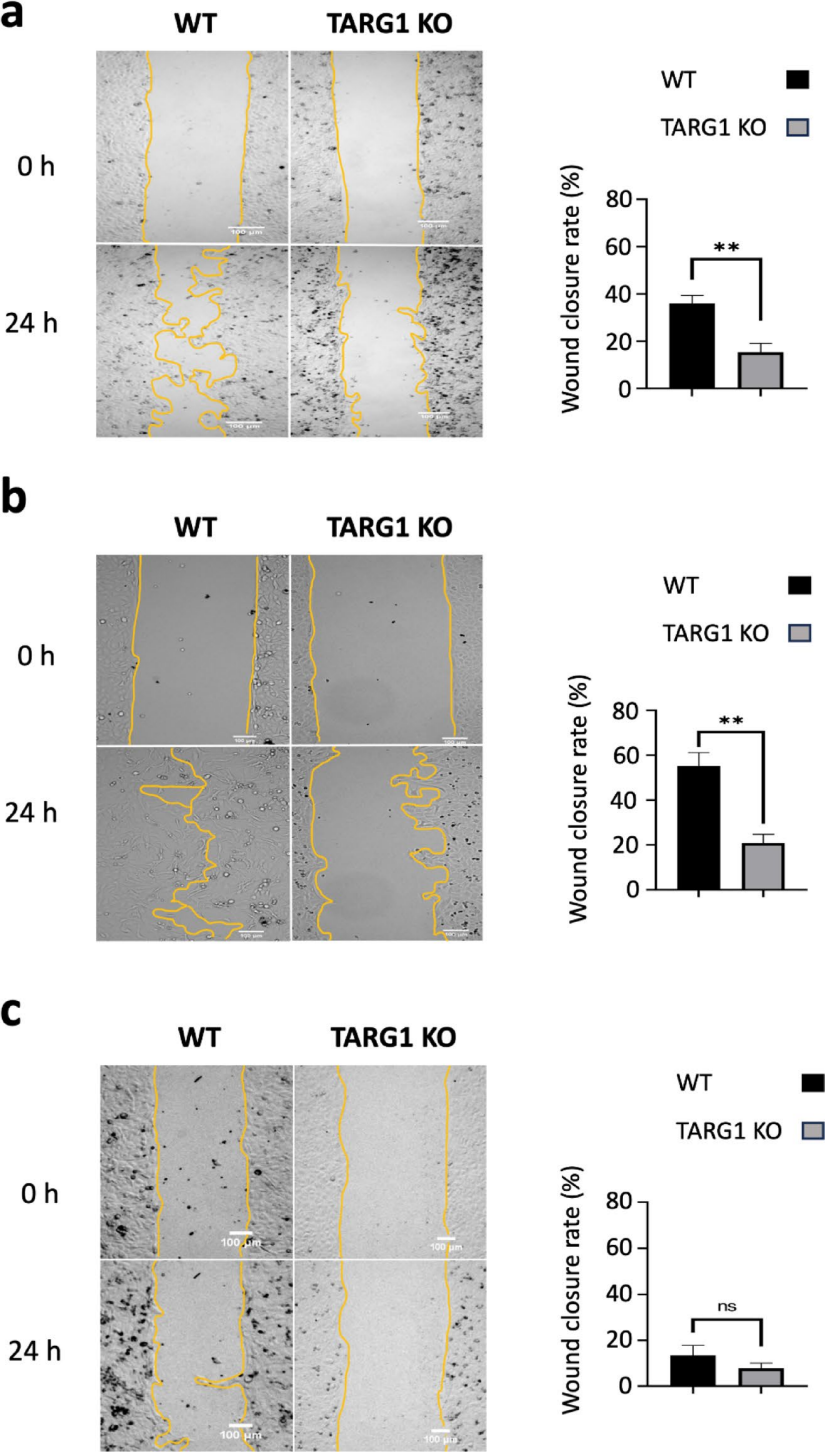
TARG1 loss leads to impaired cell migration. (**a**) Representative images (left) of wound healing assays with WT and TARG1 KO cells immediately after gap generation (0 h) and 24 h after it (24 h) in the presence of 100 ng/ml h-EGF in serum free medium, (**b**) in 10% FBS containing medium, and (**c**) in serum free medium. Scale bar, 100 μm. Wound closure rate (right) was determined as the percentage of gap closure 24 h after would generation. Data are mean ± SEM of n ≥ 3 independent experiments. Asterisks indicate p-values obtained by multiple t-test Holm-Sidak method, with alpha = 0.05. (ns. Not significant; **p < 0.01).

To assess if TARG1 deficiency has any significant impact on cell migration, we performed wound healing assay using TARG1 knockout (KO) and control, wild-type (WT) cell lines. Cells were cultured to confluence in culture wells with Ibidi inserts, which creates a uniform scratch in the monolayer after the removal of the insert. The cells were incubated in serum-free medium to minimize proliferation effects and stimulated with h-EGF to promote migration into the created gap. Wound closure was measured 24 h after the addition of h-EGF. Quantitative analysis revealed that TARG1 KO cells stimulated with h-EGF exhibited significantly reduced migration compared to WT cells, with a slower rate of wound closure (Fig. 1a). Cells stimulated with 10% FBS-containing medium served as positive control and cells kept under serum-starvation were used as negative control. The positive control yielded results comparable to those obtained with h-EGF stimulation in both cell lines (Fig. 1b), while the negative control showed no significant differences between TARG1 KO and WT indicating that the observed differences are indeed due to migration and not to different proliferative capacity of cell lines (Fig. 1c). These results suggest that TARG1 is required for efficient EGF-stimulated cell migration.

### TARG1 loss reduces EGFR protein level

**Fig. 2:**
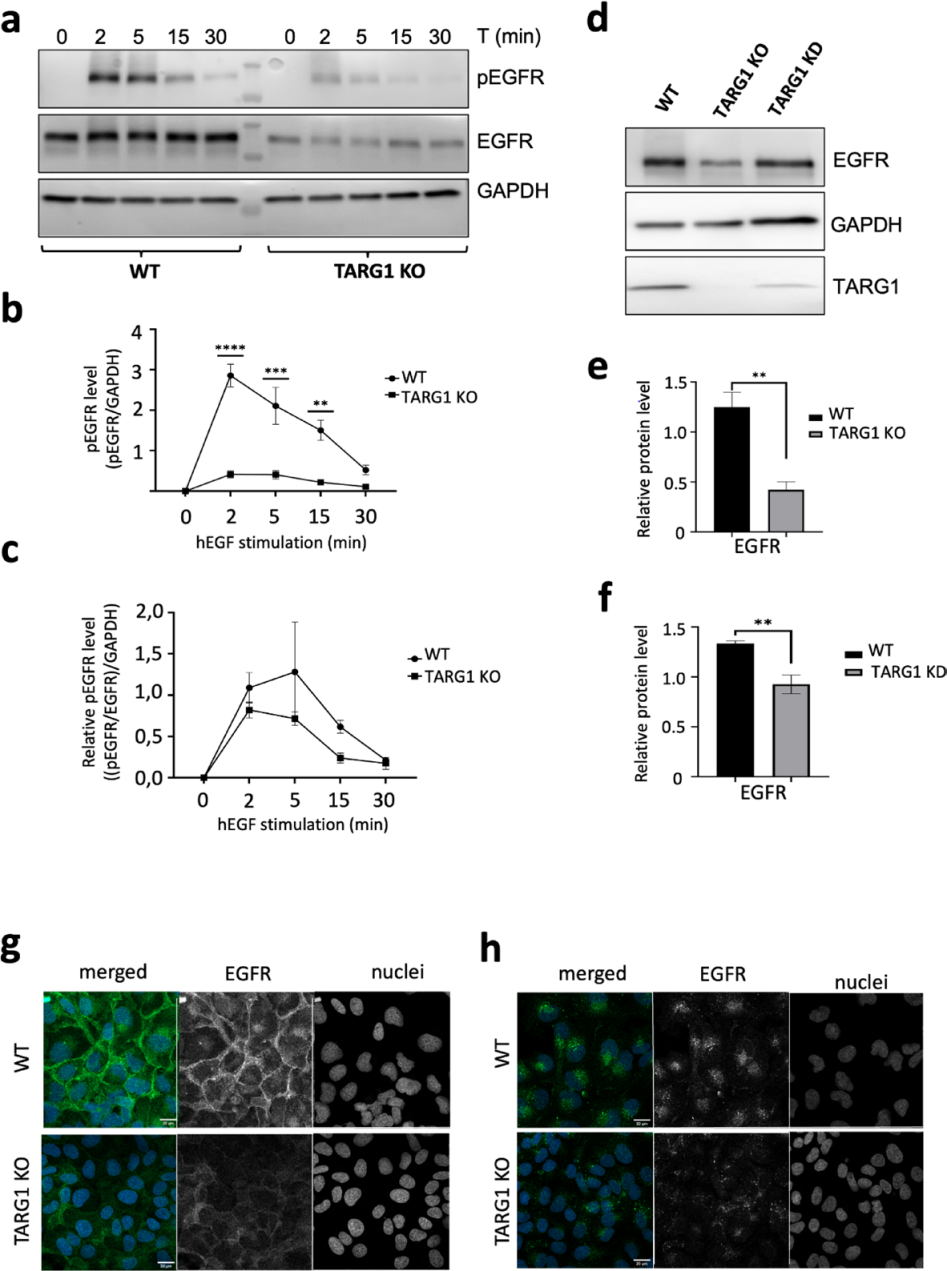
EGFR protein level is reduced in the TARG1 mutants. (**a**) Representative Western blot of phosphorylated EGFR (pEGFR), total EGFR (EGFR) and GAPDH at the indicated time points following h-EGF (100 ng/ml) stimulation in WT and TARG1 KO cells. GAPDH served as loading control. (**b**) Quantification of the phosphorylated EGFR (pEGFR) levels at the indicated time points in WT and TARG1 KO cells; (asterisks represents the significant differences between the WT and TARG1 KO at each time point) and the total pEGFR was calculated by dividing the pEGFR signal with the GAPDH signal) (**c**) Quantification of the relative phospho-EGFR (we divided the pEGFR/EGFR signal and then this relative pEGFR signal we further divided by the GADPH signal) level in WT and TARG1 KO cells. (**d**) Representative Western blot of the total EGFR level in WT, TARG1 KO and TARG1 knock down (KD) cell lines from whole cell lysate. Quantification of EGFR levels (**e**) in WT and the TARG1 KO and (**f**) in WT and TARG1 KD. Data in (**b**, **c**, **e**, **f**) are mean ± SEM (*n* ≥ 3). Asterisks indicate *p*-values obtained by multiple t-test Holm-Sidak method, with alpha = 0.05. (** *p* < 0.01; ***p < 0.001). Representative images of EGFR internalization dynamics by immunofluorescence experiments of EGFR in WT and TARG1 KO cells (**g**) 4 h after serum starvation and (**h**) after 30 min h-EGF (100 ng/ml) stimulation following the 4 h serum starvation. Scale bar, 20 μm.

The markedly reduced migration observed in the TARG1 KO cells prompted us to examine the expression level and activity state of EGFR in TARG1 KO using Western blot. Both total and phospho-EGFR protein levels were quantified from whole cell lysates of WT, TARG1 KO. The results revealed a reduced overall EGFR protein level and decreased receptor phosphorylation in the TARG1 KO cells (Fig. 2a,b). However, when normalizing the phospho-EGFR levels to total EGFR, no significant differences were observed between wild-type and TARG1 KO cells (Fig. 2c). To confirm the impact of TARG1 on EGFR levels, we performed additional experiments using stable miRNA (TARG1 KD) and transient siRNA transfections to silence TARG1 expression in wild-type cells. Both the stable miRNA-expressing TARG1 KD cell line and the siRNA TARG1 silencing led to reduced EGFR protein levels albeit to a smaller extent than in TARG1 KO (Fig. 2d–f; Supplementary Fig. S1a).

Next, we conducted receptor internalization assays to investigate whether the TARG1 KO cell line exhibited differences in EGFR internalization rates or vesicular trafficking dynamics, such as signal accumulation in specific cellular compartments or changes in signal intensity over time. After serum starvation, cells were stimulated with h-EGF, fixed, and subjected to immunostaining using an antibody specific to the intracellular domain of EGFR (Fig. 2g,h). Comparison between wild-type and TARG1 KO cells revealed no significant differences in internalization dynamics. However, TARG1 KO cells displayed reduced EGFR signal intensity. Taken together, these results suggest that the reduced EGFR phosphorylation in TARG1 mutants is associated with lower EGFR protein levels rather than altered phosphorylation efficiency. Additionally, ligand-induced EGFR endocytosis and trafficking did not differ considerably between the WT and TARG1 KO cells.

To test whether reduced EGFR levels contribute functionally to the impaired migration observed in TARG1 KO cells, we performed rescue experiments by overexpressing EGFR-GFP in both wild-type and TARG1 KO backgrounds. Cells were transfected with either an EGFR-GFP expression plasmid^33^ or a GFP only control plasmid and subjected to a wound healing assay to evaluate cell migration. Transfections provided 25–30% transfection efficiency with minimal toxicity (Supplementary Fig. S1c). Migration was assessed by imaging the wound area immediately after insert removal and again after 18 h. No increase in wound closure was observed in EGFR-GFP-transfected cells compared to GFP only controls in either the wild-type or TARG1 KO background (Supplementary Fig. S1d,e). These results suggest that EGFR overexpression under the current conditions is insufficient to enhance cell migration and does not rescue the migratory defect caused by TARG1 loss.

### RNA turnover is increased in TARG1 knockouts

**Fig. 3:**
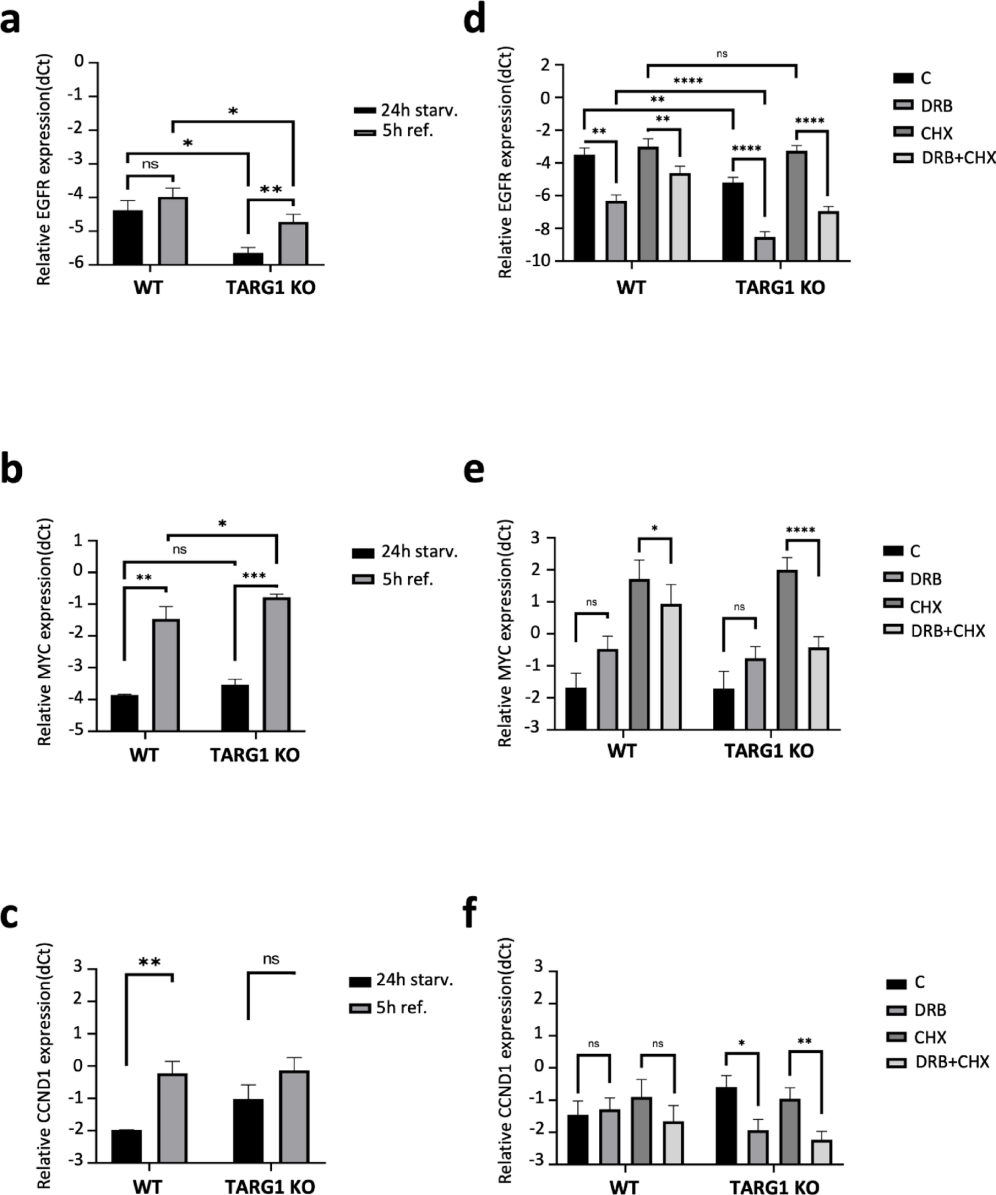
Changes in mRNA levels of EGFR and response genes (MYC, CCDN1) were revealed by qRT-PCR analysis. (**a**) EGFR, (**b**) MYC, (**c**) CCND1 mRNA level in WT and TARG1 KO cells after 24 h serum starvation (24 h starv.), and after 24 serum starvation followed by 5 h of 10% serum refeeding (5 h ref.). (**d**) EGFR, (**e**) MYC, (**f**) CCND1 mRNA levels in WT and TARG1 KO cells cultured in normal medium (black bars; C), following transcription block (medium grey bars; DRB for 12 h), following translation block (dark grey bars; CHX for 12 h), and following combined transcription and translation block (light grey bars; DRB + CHX for 12 h). Relative gene expression was calculated by subtracting the Ct value of the gene of interest from the Ct value of RPL27. Data are mean ± SEM (n ≥ 3). Asterisks indicate *p*-values obtained by two-sided two-sample unequal variance t-test. (ns. not significant; *p < 0.05, **p < 0.01, ****p < 0.0001).

To investigate whether the observed reduction in EGFR protein levels in TARG1 KO cells was accompanied by corresponding changes at the mRNA level, we measured EGFR mRNA expression in WT and TARG1 KO cells. Cells were subjected to 24-h serum starvation followed by a 5-h recovery period in medium supplemented with 10% FBS. Quantitative RT-PCR (qRT-PCR) analysis revealed that EGFR mRNA levels were significantly lower in TARG1 KO cells compared to WT cells in both conditions. Notably, while EGFR mRNA levels in WT cells did not change significantly after the 5-h recovery, a significant increase was observed in TARG1 KO cells during this period (Fig. 3a), although the serum-induced changes in gene expression were not abrogated the reduced EGFR mRNA level in the TARG1 KO cells.

To assess EGFR signaling at the gene expression level, we measured the changes in mRNA levels of two EGFR targets, MYC and cyclin D1 (CCND1) upon serum stimulation. The transcription of MYC is regulated by EGFR through the MAPK pathway^34^. The expression of CCND1 is modulated by multiple transcription factors that are downstream effectors of the EGFR signaling pathway. These include the MYC proto-oncogene and the AP-1 transcription factor complex, which is composed of Jun and c-Fos proteins^1^. The mRNA levels of MYC significantly increase in both WT and TARG1 KO upon serum stimulation (Fig. 3b). On the other hand, there was a significant increase in the mRNA level of CCND1 in wild-type cells upon serum stimulation, while in the TARG1 KO the increase was not significant (Fig. 3c). It should be noted, however, that the mRNA levels of CCND1 after serum stimulation were very similar in WT and TARG1 KO, and it was the serum-starved condition where the CCND1 mRNA level in TARG1 KO was not reduced to the level observed in WT. Altogether these results suggest that EGFR signaling is not compromised at the level of gene expression in the absence of TARG1 regardless of the reduced EGFR protein levels.

The reduced EGFR mRNA levels observed in TARG1 KO prompted us to further investigate mRNA stability and the potential roles transcription and translation in its regulation. We used Dichlorobenzimidazole 1-β-D-ribofuranoside (DRB) to inhibit RNA polymerase II-mediated transcription and cycloheximide (CHX) to block translation elongation and measured their individual and combined effects on mRNA levels of EGFR, MYC and CCND1. In normal culture medium, the mRNA levels of EGFR were significantly lower in TARG1 KO than in WT (Fig. 3d). This was further corroborated by EGFR mRNA measurements in siRNA transfected TARG1-silenced cells (Supplementary Fig. S1b). 12 h of transcription inhibition decreased EGFR mRNA level both in wild-type and TARG1 KO cells, however the reduction of EGFR mRNA was greater in TARG1 KO than in WT (Fig. 3d). While CCND1 mRNA level was lowered only in TARG1 KO (Fig. 3f). The MYC mRNA levels appear to mildly but not significantly increase in both cell lines when transcription is inhibited revealing intricate feedback between mRNA turnover and transcription. The inhibition of translation with CHX increased MYC mRNA levels in both WT and TARG1 KO (Fig. 3e). Interestingly, the difference between the EGFR mRNA levels of WT and TARG1 KO was abolished when translation is blocked, which might suggest the possibility that TARG1 acts through translational regulation. Yet, when transcription and translation was simultaneously blocked, the mRNA levels of all three tested genes dropped significantly more in TARG1 KO than in WT when compared to the CHX-only conditions (Fig. 3d–f). Altogether these results suggest that the loss of TARG1 decreased the stability of mRNAs and causes increased mRNA turnover.

### TARG1-dependent regulation of RNA distribution and translation

**Fig. 4:**
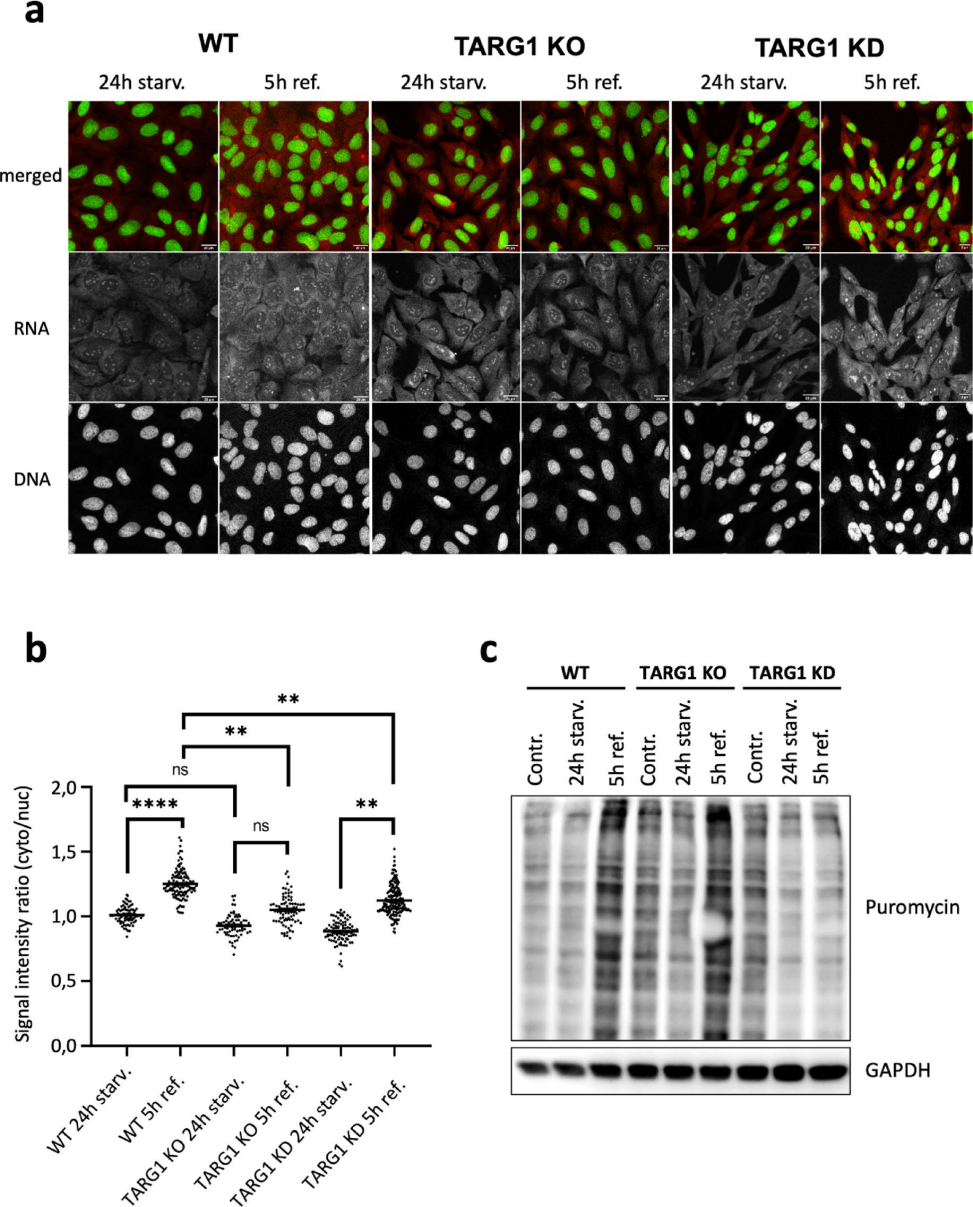
The TARG1 loss altered nuclear-cytoplasmic RNA distribution and translation after serum stimulation. (**a**) Representative images of total RNA staining in WT, TARG1 KO and TARG1 KD cells after 24 h serum starvation (24 h starv.) and after 24 h serum starvation followed by 5 h serum refeeding (5 h ref.) Scale bar, 20 μm. (**b**) Nucleo-cytoplasmic RNA distributions in WT, TARG1 KO and TARG1 KD cell lines after 24 h serum starvation (24 h starv.) and after 24 h serum starvation followed by 5 h serum refeeding (5 h ref.). Nucleo-cytoplasmic RNA distribution was quantified as the ratio of cytoplasmic to nuclear RNA intensities in individual cells, with cell and nuclear outlines identified using CellProfiler. Data are mean ± SEM (n ≥ 200). Asterisks indicate *p*-values obtained by two-way ANOVA followed by Turkey’s multiple comparison (ns. Not significant; ** *p* < 0.01; ****p < 0.0001). (**c**) SUnSET assay showing puromycin incorporation level reflecting translation rate of WT, TARG1 KO and TARG1 KD cells. GAPDH was used as loading control.

Given the supposed role of TARG1 in RNA metabolism^31^, and the observed impairment of mRNA stability in TARG1 KO cells, we aimed to investigate whether TARG1 loss affects the cellular distribution using total RNA staining (Fig. 4a). Ribosomal RNA (rRNA) accounts for approximately 80% of the total RNA, while mRNA constitutes only about 4%, alongside other functional RNAs. Ribonucleoprotein complexes, composed of rRNA and ribosomal proteins, undergo extensive maturation before forming ribosomal subunits, which requires trafficking between the nucleus and cytoplasm. Following 24 h of serum starvation and then followed by 5 h of serum stimulation, we quantified the cytoplasmic to nuclear RNA distribution by calculating the ratio of cytoplasmic to nuclear RNA intensity (Fig. 4b). In WT cells, serum stimulation significantly increased the cytoplasmic to nuclear RNA ratio, indicating a redistribution of RNA from the nucleus to the cytoplasm, which may accompany translational restart^35^. In TARG1 KO cells, we observed similar cytoplasmic to nuclear RNA distribution upon serum starvation as compared to WT cells, which increased only mildly upon the 5-h serum stimulation. In the miRNA-induced TARG1 KD the redistribution of RNA from the nucleus to the cytoplasm following serum stimulation was lower than that of WT cells and the cytoplasmic to nuclear ratio remained significantly reduced compared to serum stimulated WT. (Fig. 4b). These findings suggest that TARG1 may influence RNA metabolism, particularly processes involving ribosomal RNA, which constitutes the majority of total RNA.

To determine whether these alterations in RNA distribution were linked to changes in translation, we performed the SUnSET assay, which detects newly synthesized proteins by incorporating a brief puromycin pulse followed by anti-puromycin antibody detection^36^. As control, WT and TARG1 KO cells were treated with puromycin alone or pre-treated with the translational inhibitor CXH, and puromycin incorporation was analyzed by Western blotting. Puromycin efficiently labels newly synthetized proteins, while translational inhibition abrogates puromycin incorporation (Supplementary Fig. S2). Interestingly, the puromycin labeling revealed increased translation in TARG1 KO compared to WT.

We then examined whether serum starvation followed by serum stimulation influenced translation in WT, TARG1 KO and TARG1 KD cell lines. Under normal culture conditions, puromycin labeling was increased in both TARG1 KO and KD cell lines compared to WT. Serum starvation for 24 h had little effect on translation of WT cells, while translation in both TARG1 KO and KD declined to levels similar to WT. After 5 h of serum stimulation, translation increased in WT and TARG1 KO, as indicated by elevated puromycin labeling, but this upregulation was not observed in TARG1 KD cells (Fig. 4c). These results showed an elevated level of translation in TARG1 KO and KD compared to the WT further supporting that TARG1 plays a role in translational regulation.

### TARG1 mutant cell lines showed sensitivity against MEK inhibition

Translation and transcription are regulated by two major signaling pathways the PI3K/mTOR and Ras/Raf/MEK/ERK pathways^37^. We aimed to investigate whether cell proliferation following treatment with specific pathway inhibitors was affected by altered TARG1 expression.

**Fig. 5:**
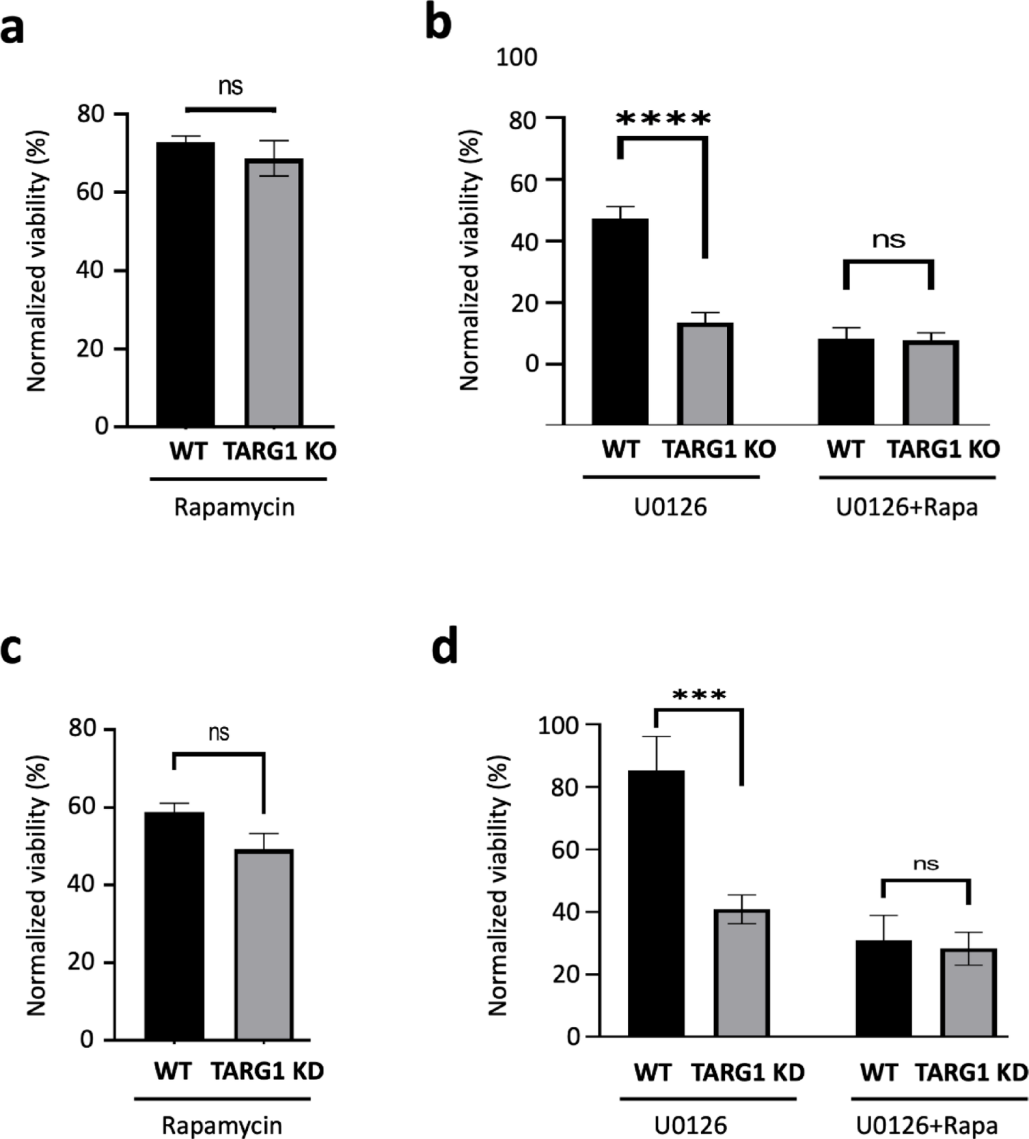
TARG1 KO cells have increased sensitivity to MEK inhibition. Cell viability assay of WT and TARG1 KO (**a**, **b**) or TARG1 KD (**c**, **d**) cells treated with 100 nM Rapamycin (**a**,** c**), and with the MEK1/2 inhibitor, 25 µM U0126 alone (U0126) or in combination with 100 nM Rapamycin (U0126 + Rapa) (**b**, **d**) for 6 days. The graphs show the relative viability normalized to the untreated samples of each genotype. Data are mean ± SEM of *n* ≥ 3 independent experiments. Asterisks indicate *p*-values obtained by multiple t-test Holm-Sidak method, with alpha = 0.05. (ns. Not significant; ***p < 0.001;****p < 0.0001).

We treated cells with rapamycin, an mTOR inhibitor and U0126, a MEK1/2 inhibitor alone or in combination. Rapamycin treatment alone did not reveal significant differences in viability between WT and TARG1 KO cells (Fig. 5a). However, U0126 reduced cell viability to a greater extent in both TARG1 KD and KO cells than in wild-type cells. (Fig. 5b,d). Notably, co-treatment with U0126 and rapamycin increased the sensitivity only in WT cells, thus eliminating the differential sensitivity of WT and TARG1KO cells to MEK1/2 inhibition (Fig. 5b,d). The sensitivity of TARG1 KD to MEK1/2 and mTOR inhibition was almost identical to that of TARG1 KO (Fig. 5c,d). These results suggest that TARG1 may influence a regulatory target involved in the crosstalk between the PI3K/mTOR and Ras/MEK/ERK pathways, potentially by modulating mTOR activity.

## Discussion

In this study, we explored the role of TARG1, a macrodomain-containing (ADP-ribosyl)hydrolase, in regulating EGFR signaling and RNA metabolism. Our findings indicate that TARG1 modulates EGFR expression and mRNA stability, suggesting that ADP-ribosylation may contribute to the regulation of the EGFR signaling pathway. TARG1 loss impaired cell migration, a key process regulated by EGFR signaling. Additionally, TARG1-deficient cells exhibited reduced EGFR mRNA and protein levels, accompanied by lower EGFR phosphorylation, suggesting that TARG1 influences both EGFR expression and activity. However, despite significantly reduced EGFR mRNA levels in TARG1 KO cells, serum-induced gene expression changes were not abrogated, indicating that TARG1 does not influence EGFR signaling at the transcriptional level. Importantly, while EGFR loss likely contributes to the observed defects in proliferation, our findings do not exclude the possibility that TARG1 deficiency disrupts additional, EGFR-independent mechanisms of cell proliferation that may indirectly impair EGFR-driven growth.

One of our findings was the increased mRNA turnover observed in TARG1-deficient cells. Specifically, TARG1 loss led to enhanced degradation of EGFR mRNA and the mRNAs of its downstream targets, MYC and Cyclin D1. These results reveal that TARG1 plays a role in stabilizing mRNA transcripts and preventing their premature degradation. Our data further indicate that TARG1 regulates mRNA stability via translational mechanisms, as inhibiting translation reversed the differences in EGFR mRNA levels between WT and TARG1 KO cells. Additionally, increased puromycin incorporation in TARG1 KO cells suggests enhanced global translation in these cells. Notably, despite this increased translational activity, EGFR protein levels were reduced in TARG1-deficient cells. However, we did not investigate whether this increase in translation resulted in higher protein synthesis overall or led to the production of aberrant proteins. Altogether, these results imply that TARG1 may influence mRNA processing and stabilization by modulating translation efficiency.

These observations align with previous studies identifying numerous TARG1-interacting proteins involved in RNA biogenesis, including enzymes associated with ribosomal maturation, RNA splicing, nuclear export, and translational machinery^31^. Given the functional relevance of these proteins in RNA regulation, it is plausible that TARG1, through its (ADP-ribosyl)hydrolase activity, modulates RNA processing, impacting mRNA stability, maturation, and translation. However, it remains unclear whether these effects depend solely on TARG1’s catalytic activity, as catalytically inactive TARG1 has also been shown to bind RNA^38^.

Several PARPs have been reported to bind RNA through conserved CCCH RNA-binding domains (PARP7, PARP12 and PARP13) or RRM motifs (PARP10 and PARP14)^39^ highlighting their potential influence on RNA-related processes^40^. The absence of TARG1 may amplify these effects. Moreover, PARP1 has also been shown to modulate mRNA biogenesis^41^. During thermal stress, PARP1 PARylates poly(A) polymerases, causing their dissociation from RNA and leading to a global reduction in polyadenylation. This, in turn, can impair RNA stability, hinder mRNA export, and reduce translation efficiency^42^. Additionally, PARP1 depletion has been linked to changes in EGFR expression^43^. Beyond nuclear PARP1, an ER transmembrane mono(ADP-ribose)transferase, PARP16, has been shown to MARylate ribosomal proteins essential for polysome assembly, thereby regulating translation initiation^44^. Interestingly, ERK signaling—known to regulate ribosomal proteins independently of mTOR—targets ribosomal subunits such as RPS6^45^, which are also substrates of PARP16^44^.

A recent study reported that TARG1 depletion affected the regulation of a ribosome- associated protein, RACK1 MARylation, increasing the translation of certain RNAs while reducing that of others in OVCAR3, an ovarian cancer cell line^46^. In contrast, a previous study in TARG1-deficient HeLa cells did not report changes in proliferation or translation^38^, suggesting that TARG1’s role in RNA metabolism may be cell type-dependent.

Notably, nucleic acids—including both DNA and RNA—can also undergo ADP-ribosylation^28^ and MARylation of the 5’-terminal phosphate of RNA has been shown to inhibit translation and affect mRNA stability^47^.

Together, these findings underscore the numerous ways in which ADP-ribosylation regulates RNA metabolism—both indirectly through RNA-binding proteins and translation factors, and directly through RNA modifications—highlighting its broad influence on gene expression and cellular signaling.

Our study demonstrated that TARG1 deficiency significantly reduced EGFR expression, a key proto-oncogene involved in cancer-related processes such as migration, proliferation, and adhesion^8^. Many current therapeutic strategies target EGFR by inhibiting its kinase activity or preventing extracellular ligand binding. However, resistance-associated mutations often reduce drug efficacy^5,11^. Our findings suggest a novel regulatory mechanism in which TARG1 modulates EGFR expression at the mRNA level, potentially offering a new therapeutic avenue to circumvent EGFR mutation-driven drug resistance.

Furthermore, the increased sensitivity of TARG1-deficient cells to MEK1/2 inhibition suggests a potential role for TARG1 in signaling pathway regulation. MEK1/2, a key component of the Ras/Raf/MEK/ERK pathway, is critical for cell survival and proliferation^48,49^. This pathway is implicated in approximately one-third of all cancers due to its central role in gene expression, cell proliferation, survival, and apoptosis^50^. As a result, MEK inhibitors have been extensively studied as potential cancer therapies. However, despite promising results, not all patients respond to these inhibitors, and resistance frequently develops in those who initially do^50^. Our results suggest that TARG1 may mediate crosstalk between the Ras/MEK/ERK and PI3K/mTOR pathways. The heightened sensitivity of TARG1-deficient cells to MEK1/2 inhibition raises the possibility that TARG1 could be a novel therapeutic target for enhancing the efficacy of MEK inhibitors in cancer therapy.

The role of ADP-ribosylation in RNA metabolism, particularly in mRNA maturation and translation, is an emerging area of investigation. Given the clinical relevance of EGFR signaling in cancer, understanding TARG1’s function could have significant therapeutic implications, particularly in tumors reliant on EGFR-driven proliferation and migration. Future studies should aim to elucidate the precise molecular mechanisms by which TARG1 modulates RNA metabolism and signaling pathways and explore its potential as a therapeutic target in cancer treatment.

## Materials and methods

### Cell lines

U2-OS (HTB-96, ATCC) wild type and TARG1 knock out (CRISPR/Cas) cell lines have been described previously^51^ and were cultured in DMEM (LM-D1109 Dulbecco’s Modified Eagles High Glucose w/ L-Glutamine w/o Sodium Pyruvate, Biosera Cholet, France), supplemented with 10% FBS (FB-1090/500 Fetal Bovine Serum (South America) Biosera Cholet, France), 1 × NEAA (E1154 MEM, Biosera Cholet, France) and Penicillin/Streptomycin (A4118, Biosera Cholet, France) at 37 °C in a humidified cell incubator with 5% CO_2_. The cell lines were routinely tested for mycoplasma contamination using a qPCR-based approach (MQ-50 MycoQuant Mycoplasma Quantification Kit AVIDIN, Szeged, Hungary). For knockdown of TARG1, we used a stable U2-OS cell line constitutively expressing miRNA targeting TARG1. The stable TARG1 knockdown U2-OS cell line was created by genome integration of a transposon-based vector, pNeo-miR constitutively expressing amiR targeting GCCCACTGTATCAGTGAGGATT sequence of TARG1 mRNA. This approach was adapted from the methods described ealier^52^. Briefly, amiR elements were designed following the miR-E backbone structure, and the guide sequences were selected based on their target specificity as previously reported^53^. The amiR sequences were incorporated into the AgeI/XbaI sites of pNeo-miR. This vector contains Sleeping-beauty (SB) transposon elements for stable integration and a Neomycin expression unit. For the selection of genome-integrated clones, 800 µg/ml G418 (HY-17561, MedChemExpress, Monmouth Junction, NJ, USA) was used for three weeks. For the transient siRNA transfections, ON-TARGETplus, SMARTpool Human OARD1 siRNA (Horizon Discovery; Dharmacon™ Reagents; Catalog ID: L-015886–02-0005) to target TARG1, Ambion™ Silencer™Select Human C20orf133 (s44382,s4480 Ambion, Thermo Fisher Scientific, Waltham, US) for MacroD2 and ON-TARGETplus Non-targeting Control siRNA #1 (Horizon Discovery; Dharmacon™ Reagents; Catalog ID: D-001810-01-20) as control were used.

The cells were transfected with Screenfect siRNA transfection reagent (ScreenFect; Cat#S-4001), following the manufacturer instructions, then 72 h following transfection lysates were collected for analysis.

### Western blot

The cells were seeded at cell numbers to reach 70–80% confluency for the treatments. In case of basal condition blots, the cells were collected right after they reached confluency. For phosphor-EGFR signal detection, FBS was withdrawn for 4 h and 100 ng/ml h-EGF (E9644 Sigma Aldrich Saint Louis MO US) containing medium was added back to the cells until the indicated timepoints of sample collection. Cell lysates were collected in 4% SDS lysis buffer (4% SDS, 150 mM NaCl, 5 mM MgCl_2_, 50 mM HEPES, pH 7.4). The lysates were spun down at 13.000 rpm for 25 min and the protein concentration of supernatants was determine using NanoDrop 2000™ spectrophotometer (Thermo Fisher Scientific Inc,). Lysates with equal protein amount were resolved on 9% TRIS/Glycine SDS-PAGE gel and blotted onto nitrocellulose (GE10600004 Amersham Protran Premium 0.2 NC, Cytiva, Boston, MA, USA) or PVDF (GE10600021 Amersham™ Hybond® P, Cytiva, Boston, MA, USA membrane in 10% methanol containing transfer buffer. The blotting efficacy was checked with Ponceau S staining. The membranes were blocked either with 4% gelatin (G7765, Sigma Aldrich Saint Louis MO US) for phospho blots or 5% BSA (A7906 Sigma Aldrich Saint Louis MO US) for 1 h in PBST (1 × PBS, 0.05% Tween-20). After blocking at room temperature (RT), the membranes were incubated with the primary antibodies: anti-EGFR [EP38Y] antibody (ab52894 Abcam, Cambridge, UK, 1:000), anti-pEGFR [phospho Y1068] (ab32430, Abcam Cambridge, UK, 1:8000), anti-GAPDH antibody (PA1-16,777, Thermo Fisher Scientific Inc., 1:3000) and anti-TARG1 antibody (25249-1-AP, ChromoTek GmbH, Planegg-Martinsried, Germany, 1:2000) overnight at 4 °C. After washing, the secondary antibody (G-21234 Goat anti-Rabbit IgG (H + L) Secondary Antibody, HRP Thermo Fisher Scientific Inc.1:10.000) was added in blocking buffer for 1 h at RT. The protein bands were visualized with enhanced chemiluminescence (ECL) solution (SuperSignal™ West Pico PLUS Chemiluminescent Substrate, 34,580 Thermo Fisher Scientific Inc.) using Alliance Q9 Advanced imaging system (Uvitec Cambridge,UK). The intensity of the signals was measured with ImageJ (ImageJ, U.S. National Institutes of Health, Bethesda, MD, USA) and normalized to the loading control signal intensity.

### Wound healing assay

One day before the experiment, cells were seeded into a well of micro-insert 4-well system as recommended by the manufacturer [3 × 10^5^ cells/ml in a total volume of 70 µl end volume^54^], (80469 Culture-Insert 4-Well ibidi GmbH, Gräfelfing, Germany). The inserts were removed, and the cells were washed with 37 °C DMEM (LM-D1109 Biosera Cholet France) without FBS before being cultured under the indicated conditions: serum-free medium, complete medium, or serum-free medium containing 100 ng/ml h-EGF. Cell migration was monitored at 37 °C using a Zeiss Cell Discoverer 7 fluorescence microscope (Zeiss, Jena, Germany) with CO_2_ levels regulated at 5%. Images were taken every 30 min for 24 h from the same areas. The closure rate of the gap between the cells was calculated using the following formula: *wound closure rate (%)* = *[(0 h—24 h) / 0 h]* × *100*, where “0 h” was the cell-free area of the gap at the start of imaging, and “24 h” represents the same measurement at the final time point of the experiment. Measurements were performed using ImageJ (ImageJ, U.S. National Institutes of Health, Bethesda, MD, USA).

To overexpress the EGFR in WT or TARG1 KO cells transient transfection was performed with the EGFR-EGFP or pEGFP-C1 (Invitrogen) plasmids. EGFR-GFP was a gift from Alexander Sorkin (Addgene plasmid #32,751)^33^. 3 × 10^5^ cells were seeded into a 35 mm dish and next day a complex of 3 µg plasmid and 15 µl TransIT®-LT1 Transfection Reagent (MIR 2300, Mirus Bio, Madison, USA) was added to the cells according to manufacturer instruction. After 3 days, the cells were trypsinized, and 4 × 10^4^ cells in a total volume of 110 µl were seeded into each well of a Culture-Insert 4-Well (ibidi GmbH, Gräfelfing, Germany), and the remaining cells were used in flow cytometry analysis to determine their transfection efficiency. The samples were analyzed with CytoFLEX S flow cytometer (Beckman Coulter Life Sciences). The measurements were evaluated using Kaluza Analysis software (Beckman Coulter Life Sciences).

The next day, the inserts were removed, and the cells were washed with 37 °C DMEM and starved for 4 h before complete medium was added. Cell migration was monitored for 18 h. Pictures were taken with an Olympus digital camera mounted on an inverted microscope with a 10 × objective after the removal of the insert (0 h) and 18 h (18 h) later. The closure rate of the gap was calculated as described above except here the endpoint was at 18 h.

### EGFR internalization assay

Cells were seeded on coverslips and allowed to grow until confluency. Culture medium was changed for 4 h to serum-free DMEM then supplemented with 100 ng/ml h-EGF for 30 min. After washing with PBS, the cells were fixed with 4% paraformaldehyde for 10 min at room temperature. Next, PBS containing 0,2% TritonX-100 was added for 10 min for permeabilization. Following blocking with PBS supplemented with 0,1% Tritonx-100 and 5% FBS for 1 h at room temperature, the cells were probed with anti-EGFR [EP38Y] antibody (ab52894 Abcam, Cambridge, UK, 1:000) in blocking buffer overnight at 4 °C. Subsequently, the cells were washed 3 times with PBS 0,1% Triton X-100 for 5 min, then probed with Goat anti-Rabbit IgG (H + L) Cross-Adsorbed Secondary Antibody, Alexa Fluor™ 488, (A11008 Invitrogen, Thermo Fisher Scientific Inc., 1:500) for 1 h on room temperature. Following washes, the nuclei of cells were counterstained with Hoechst 33,342 (H3570 Thermo Fisher Scientific Inc., 1:10,000). After mounting with Prolong™ Glass Antifade Mountant (P36982 Thermo Fisher Scientific Inc.) the images were acquired with Zeiss LSM 800 confocal microscope, Plan-Apochromat, 40X/0.95 NA and 20X/0.8 NA air objective, Fluorescent – LSM, GaAsP (Gallium Arsenide) PMT detector using the Zen 2.6 software.

### qRT-PCR

To ensure growth restricted condition, cells were serum starved for 24 h or serum starved for 24 h and further cultured in 10% serum containing DMEM for 5 h before RNA preparation. To investigate the effects of transcription and translation blocks cells were treated with 75 µM 5,6-Dichlorobenzimidazole 1-β-D-ribofuranoside (DRB, D1916 Sigma-Aldrich Saint Louis MO US) or/and 40 µg/ml cycloheximide (CHX, C7698, Sigma-Aldrich Saint Louis MO US) for 12 h. Total RNA was isolated using NucleoSpin RNA Kit (740955 Macherey–Nagel) following the manufacturer’s instructions. RNA concentration was measured using NanoDrop 2000 Spectrophotometer (Themo Fisher Scientific), and cDNA was synthesized from 1 μg of total RNA using the RevertAid First Strand cDNA Synthesis Kit (K16 22 Thermo Fisher Scientific Inc.). Each qPCR reaction contained 400 nM of the respective forward and reverse primers, 20 times diluted cDNA in 1 × SYBR Select Master Mix for CFX (4,472,953 Thermo Fisher Scientific Inc.). The used primers were:

- EGFR: fwd: 5′-GACTGCTGCCACAACCAGT-3′
  rev: 5′-CGTGGCTTCGTCTCGGAAT-3′
- MYC: fwd: 5′-AGCGACTCTGAGGAGGAACAA-3
  rev: 5′-CTTCAGACCATTCTCCTCCGG-3′
- CCND1:fwd: 5′-CCTGTCCTACTACCGCCTCA
  rev: 5′-CAGTCCGGGTCACACTTGA
- RPL27: fwd: 5′-CGCAAAGCTGTCATCGTG-3.
  rev: 5′-GTCACTTTGCGGGGGTAG-3′.

qPCR was carried out at 95 °C for 2 min, followed by 95 °C for 5 s, and annealing and extension at 60 °C for 20 s for 40 cycles in Rotor-Gene Q 2Plex (Qiagen, Hilden, Germany). The Ct values were calculated with the Rotor-Gene Q Series software 2.3.1 version. The relative expression levels were plotted using the equation: dCt = Ct_RPL27_ –Ct_GOI_. Means and error bars were calculated in Microsoft Excel and derive from three independent biological replicates.

### Total RNA staining

Cells were seeded on coverslips. From following day cells were serum starved for 24 h and then reconstituted with 10% serum containing DMEM for 5 h or left in serum-depleted DMEM (in the case of 24 h samples). Total RNA was visualized with the Cell Navigator Live Cell RNA Imaging Kit (AAT Bioquest Pleasanton, CA, US) according to the manufacturer’s instructions. StrandBrite™ RNA Green, used in this kit, exhibits excellent RNA selectivity. DNA was stained with Hoechst 33342 (H3570 Thermo Fisher Scientific Inc.) diluted in PBS (1:10.000) Pictures were taken with the Zeiss LSM 800 confocal microscope, Plan-Apochromat, 40X/0.95 NA and 20X/0.8 NA air objective and GaAsP (Gallium Arsenide) PMT detector using the Zen 2.6 software. The nucleo-cytoplasmic RNA intensity ratio was measured with the open-source cell image analysis software CellProfiler using a custom pipeline. Briefly, the area of the nucleus was segmented based on the Hoechst channel. Next, the cell outlines were defined by propagation starting from the segmented nuclei using the RNA channel. The cytoplasms were identified as the propagated cytoplasmic areas minus the area of the nucleus. To calculate the nucleo-cytoplasmic RNA intensity ratio the mean intensities of the RNA channel in the cytoplasmic and nuclear areas were measured, and the mean cytoplasmic RNA intensity was divided by the corresponding mean nuclear RNA intensity for each segmented nucleus. The data were plotted, and the statistical tests were done using GraphPad Prism (GraphPad Software, Boston, Massachusetts USA, www.graphpad.com).

### SUnSET assay for detection of protein synthesis

U2-OS wild type, TARG1 knock out and stable TARG1 knockdown cells were cultured in normal culture medium or under serum withdrawal for 24 h, and then the indicated samples were serum stimulated for additional 5 h. Protein synthesis was detected with SunSET assay^36^. Briefly, 1 µM puromycin (sc-108071C, Santa Cruz Biotechnology, Dallas, TX, USA) was added to cell cultures and incubated for 30 min. For negative control, the samples were pre-treated with 100 µg/ml cycloheximide (C7698, Sigma-Aldrich Saint Louis MO US) for 10 min prior adding puromycin. After puromycin-treatment the cells were washed with PBS and lysed with 4% SDS lysis buffer and protein concentrations were determine using NanoDrop 2000™ spectrophotometer (Thermo Fisher Scientific Inc,). Equal amounts of protein were separated on 10% SDS-PAGE and transferred to nitrocellulose membrane. The membranes were blocked with 3% gelatin in PBST and incubated with anti-Puromycin mouse monoclonal antibody (MABE343, Sigma-Aldrich Saint Louis MO US 1:20,000), followed by HRP-conjugated goat anti-mouse IgG (H + L) (31,432, Invitrogen Thermo Fisher Scientific Inc., 1:10,000). The protein bands were visualized with ECL solution (SuperSignal™ West Pico PLUS Chemiluminescent Substrate, 34,580 Thermo Fisher Scientific Inc.) using Alliance Q9 Advanced imaging system (Uvitec Cambridge,UK). GAPDH was used as loading control.

### Cell proliferation assay

For the cell proliferation assays cell lines were treated with Rapamycin (37,094 Vetranal analytic standard, Merck KGaA, Darmstadt, Germany) and U0126 (9903, Cell Signaling Technology Inc. Danvers MA US)0.1000 cells were seeded in each well of 96-well plates and the next day 100 ng/ml Rapamycin, 25 μM U0126 or a combination of these were administered in DMEM supplemented with 10% FBS. After 72 h the culture medium was changed to a fresh one for an additional 72 h. The concentrations of the drugs were kept the same during the experiment (6 days). On the 6^th^ day culture medium was replaced with Gibco™ Leibovitz’s L-15 Medium, no phenol red (11540556, Thermo Fisher Scientific Inc.) containing 25ug/ml Resazurin (199303 Sigma Aldrich Saint Louis, MO, US) and incubated for 30 min in a CO_2_ thermostat. The fluorescent metabolic product was measured using a Bio-Tek Synergy H1 (Agilent Technologies Santa Clara, CA US) microplate reader with a 530/590 filter set. The viability of each sample was normalized to the untreated samples of the corresponding genotype.

### Statistical analysis

Results were expressed as mean ± SEM from at least 3 biological replicates in each assay. Statistical significance determined as it is described in figure legends (*p* = *0.05* was taken, as a significant difference in each analysis).

## Electronic supplementary material

Below is the link to the electronic supplementary material.

Supplementary Material 1

## Data Availability

The data generated during the current study will be shared on reasonable request to the corresponding authors.
