# Supplementary material for: TARG1 affects EGFR signaling through the regulation of RNA metabolism
Source: Sci Rep. 2025 Jul 2;15:23651. doi: 10.1038/s41598-025-08010-5 (PMC12222879; doi:10.1038/s41598-025-08010-5)

Supplementary Information

TARG1 affects EGFR signaling through the regulation of RNA metabolism

Mihály Mérey[1, 2], Roberta Fajka-Boja[1, 3], Gergely Imre[4, 5], Péter Gudmann[6], Zsolt Török[6], Lajos Mátés[4], Ágnes Czibula[1, 3, *], Gyula Timinszky[1, *]

1 Laboratory of DNA Damage and Nuclear Dynamics, Institute of Genetics, HUN-REN Biological Research Centre, 6726 Szeged, Hungary

2 Doctoral School of Multidisciplinary Medical Sciences, University of Szeged, 6720 Szeged, Hungary

3 Department of Immunology, University of Szeged, 6720 Szeged, Hungary

4 Laboratory of Cancer Genome Research, Institute of Genetics, HUN-REN Biological Research Centre, 6726 Szeged, Hungary

5 Doctoral School of Biology, University of Szeged, 6720 Szeged, Hungary

6 Laboratory of Molecular Stress Biology, Institute of Biochemistry, HUN-REN Biological Research Centre, 6726 Szeged, Hungary.

* Correspondence should be addressed to Á.C. (czibula.agnes@brc.hu) or G.T. (timinszky.gyula@brc.hu)

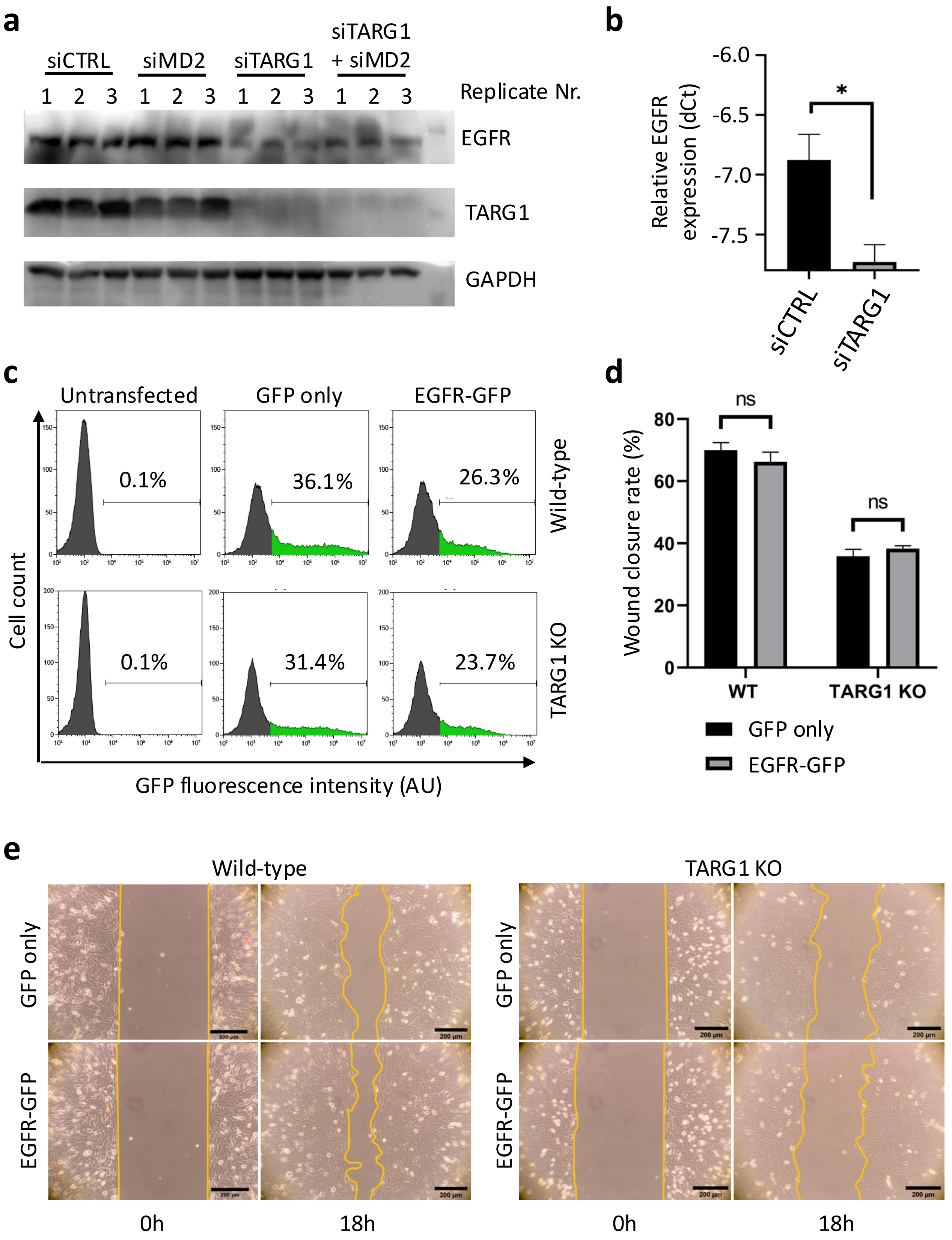

Supplementary Figure 1

Supplementary Figure 1: (**a**) Western blot analysis of U2-OS cells transfected with control siRNA (siCTRL), TARG1 siRNA (siTARG1), MacroD2 siRNA (siMD2), and the combination of siRNAs targeting both MacroD2 and TARG1 (siTARG1+siMD2). Three biological replicates were analyzed for all conditions (Replicate Nr: 1-3). EGFR and TARG1 proteins were detected by specific antibodies. GAPDH was used as loading control. (**b**) qRT-PCR analysis of the changes of EGFR mRNA level in siRNA transfected wild-type cells relative to RPL27. Control siRNA transfection (siCTRL) and TARG1 siRNA transfection (siTARG1). Data are mean ± SEM (n ≥ 3). Asterisks indicate p-values obtained by two-sided two-sample unequal variance t-test. (* $p<0.05$). (**c**) Wild-type or TARG1 KO cells were transfected with GFP only or EGFR-GFP, and the GFP fluorescence intensity was detected with flow cytometry 18 hours post-transfection. Numbers indicate the percentage of GFP positive cells. (**d**) Wound closure rate was determined as the percentage of gap closure 18 hours after would generation. Data are mean ± SEM of n ≥ 3. Multiple t-test Holm-Sidak method, with alpha = 0.05. (ns. Not significant). (**e**) Representative images of wound healing assays with WT and TARG1 KO cells transfected with GFP only control or EGFR-GFP immediately after gap generation (0 h) and 18 hours after it (18 h) in 10% FBS containing medium. Scale bar, 200 μm.

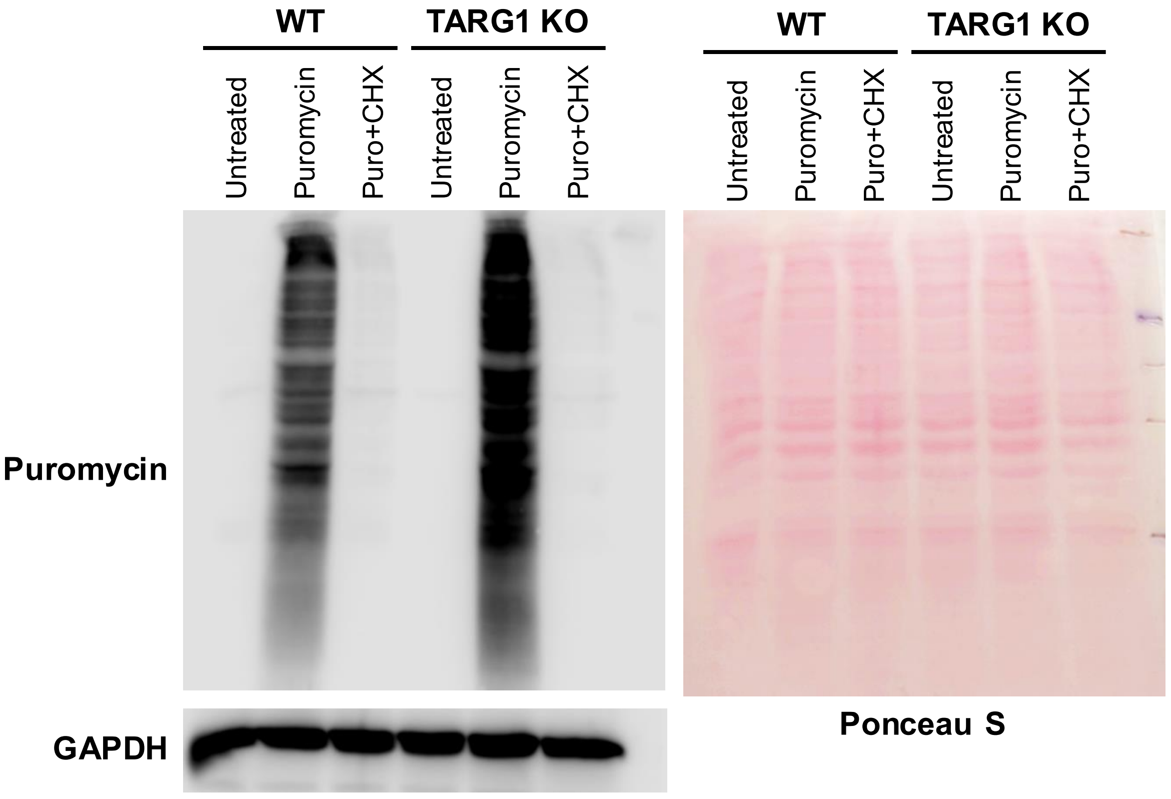

Supplementary Figure 2. U2OS WT and TARG1 KO cells were incubated with puromycin alone or with cycloheximide (Puro+CHX) for 30 min. The incorporated puromycin was detected with Western blotting. GAPDH and Ponceau S staining were used as loading controls.

Unedited Western Blot images. Regions used in the figures are indicated by dashed red boxes.

to **Figure 2b**

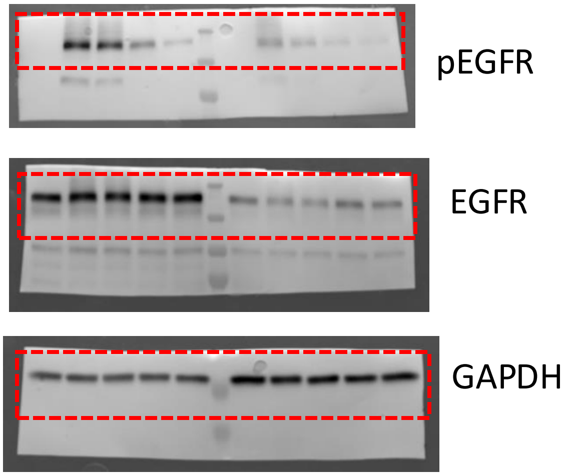

to **Figure 2d**

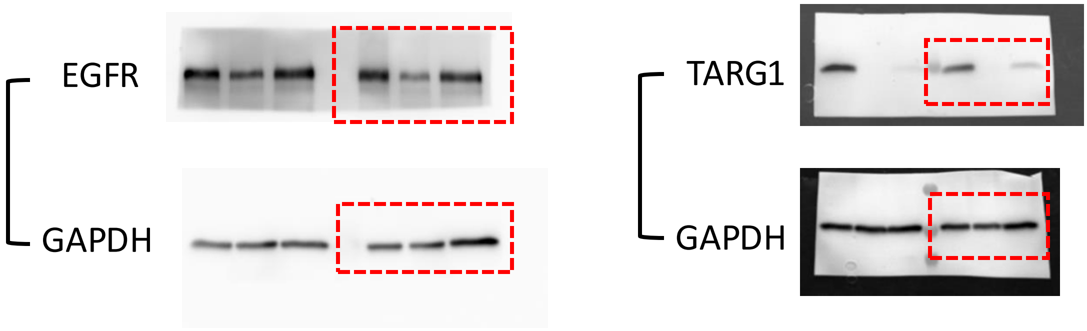

to **Supplementary Figure 1**

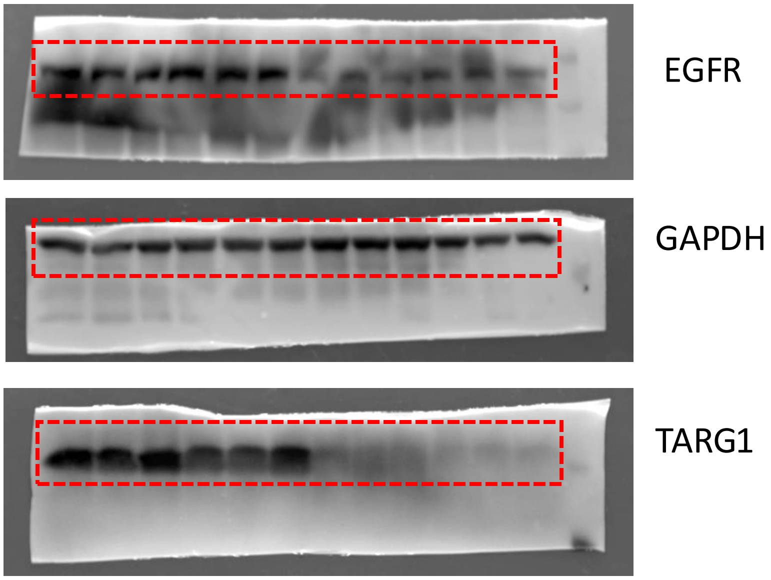

Unedited Western Blot images. Regions used in the figures are indicated by dashed red boxes.

to **Figure 4c**

Puromycin

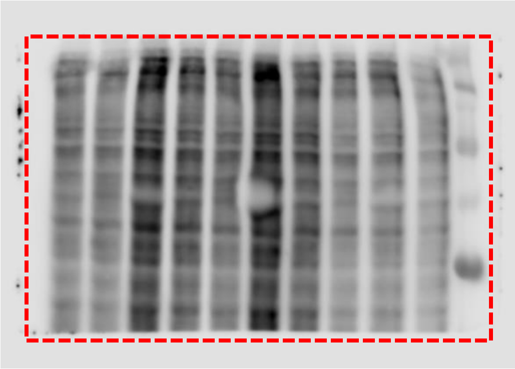

GAPDH

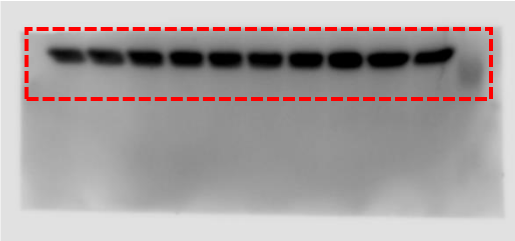

to **Supplementary Figure 2**

Puromycin

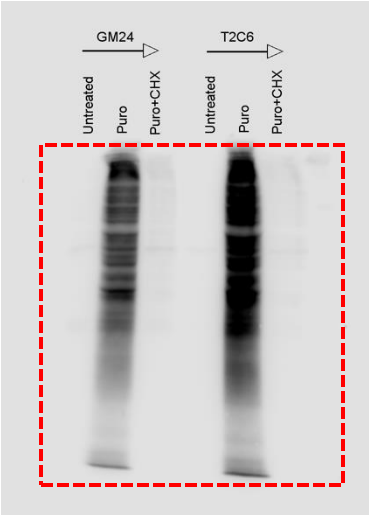

GAPDH

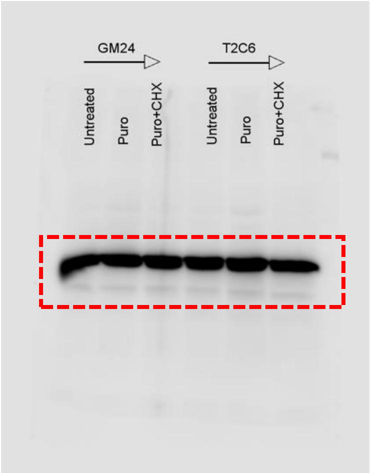

Supplement: Supplementary file 1 — Supplementary Material 1 [file 41598_2025_8010_MOESM1_ESM.pdf]
